# New Data on the Messapian Necropolis of Monte D’Elia in Alezio (Apulia, Italy) from Topographical and Geophysical Surveys

**DOI:** 10.3390/s19163494

**Published:** 2019-08-09

**Authors:** Giovanni Leucci, Lara De Giorgi, Immacolata Ditaranto, Francesco Giuri, Ivan Ferrari, Giuseppe Scardozzi

**Affiliations:** Consiglio Nazionale delle Ricerche—Istituto per i Beni Archeologici e Monumentali, Monteroni road, c/o Campus Universitario, 73100 Lecce, Italy

**Keywords:** GPR, topographical survey, 3D laser scanning, archaeological map, drone, Messapian necropolis, Alezio

## Abstract

The Messapian necropolis of Monte D’Elia is related to one of the most important ancient settlements in the Salento Peninsula (in south Italy). In order to understand the extension and layout of this necropolis in the various periods of its use, a ground-penetrating radar (GPR) prospection was undertaken in some important sample areas by a team of the Institute for Archaeological and Monumental Heritage of the National Research Council of Italy. The analysis of the GPR measurements revealed many anomalies that could be ascribed to archaeological structures (tombs), as well as other anomalies of presumable natural origin or referable to modern features. The data collected were georeferenced in the digital archaeological map of the site and integrated with a virtual reconstruction of the surveyed area.

## 1. Introduction

The present study concerns the Messapian necropolis of Monte D’Elia, located about 300 m south of the ancient settlement named Aletium (Aletion). It was inhabited between the late Iron Age and the Roman-Imperial era and lies where modern Alezio stands. The necropolis, used between the 6th and the 2nd century BC, was partially investigated between 1981 and 1985 by archaeological excavations, but the discovered tombs were subsequently buried. Later, a part of the excavated areas was again brought to light and the necropolis became part of an archaeological park. A systematic study of the necropolis does not yet exist: the site, not contain modern overlapping, is very important for the knowledge of Aletium during the Messapian period (in particular between the 6th and the 3rd century BC), when it was one of the main centres of southern Salento. Indeed, the almost complete overlapping of the modern town on the ancient settlement and most of its necropolises greatly limits the reconstruction of the various phases of historical development of Aletion/Aletium, as demonstrated by the recent archaeological map of the site [1] carried out by the Institute for Archaeological and Monumental Heritage of the National Research Council (IBAM-CNR) of Italy. Only part of the grave goods has been studied and neither the extension nor the topographical articulation of the necropolis of Monte D’Elia is known, since archaeological excavations have only partially investigated it.

The present research is based on the integration of different survey methodologies: (i) the topographical survey of the tombs still visible, also by means of 3D laser scanning techniques; (ii) the production of a large scale archaeological map of the funerary area, starting from ortho-images taken from a drone and thanks to the vectorization and the georeferencing of the old plans drawn in the 1980s, which documented the tombs excavated in 1981–1985 and now buried; (iii) geophysical surveys carried out by means of the Ground Penetrating Radar technique both on areas already affected by the excavations of 1981–1985 and now filled in, and on areas never investigated; (iv) the geophysical and archaeological interpretation of GPR measurements also thanks to the georeferencing of the results on the new topographical map. The aim of this integrated study was the reconstruction of the extension of the necropolis and its topographic articulation during the two main phases of use, the Archaic period and the Late-Classical/Hellenistic age.

## 2. Study Area

**Figure 1:**
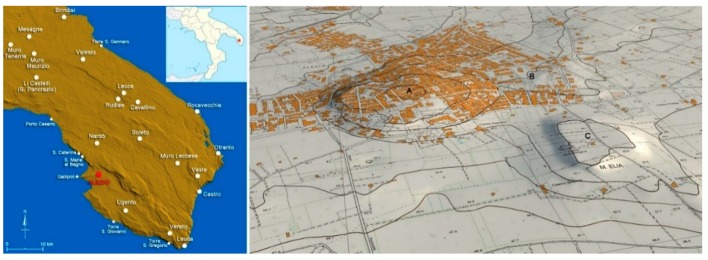
Localisation of Alezio on DTM of Salento and altimetric model of Alezio: A, hill of Lizza; B, Raggi area; C, Monte D’Elia area.

The ancient settlement of Aletion/Aletium occupied the hill today dominated by the medieval Church of S. Maria della Lizza (m 73 a.s.l.) and the less elevated area located immediately to the east, named Raggi (m 65 a.s.l.). The modern Alezio extends over both areas and partly occupies the areas further north. The site overlooks the fertile coastal plain and is located in an area of adequate height controlling the landing of Gallipoli, just 7 km away (Figure 1). The oldest literary sources concerning this ancient settlement date back to the 1st and 2nd centuries AD and documents only its Greek and Latin names Αλέτιον/Aletium. Therefore, the knowledge of the ancient phases of the settlement comes exclusively from archaeological data that offer a documentation very conditioned by the nature of the findings, mostly random and not based on systematic research [2,3,4,5,6]. The archaeological discoveries, carried out between the 19th and 20th centuries, and in particular from 1950s, were mostly fortuitous and took place after the extension of the modern town [1]. Many of these findings are epigraphes dated between the 6th and 2nd centuries BC and Aletion/Aletium has returned the highest number of inscriptions in the Messapian language among the ancient settlements of Salento [7]; most of them are funerary and many were found in the necropolis of Monte D’Elia.

The ancient settlement of Aletion/Aletium was continuously inhabited from the 7th century BC up to the Middle Ages. It is extremely difficult to read the topography of the inhabited area of the Messapian and Roman times because of the progressive overlapping of the modern town on the old settlement. The oldest archaeological evidence from the Alezio area dates back to the late Iron Age. It is likely that at least since the 7th century BC on the hill of Lizza, and probably also in the contiguous Raggi area, there was a village of huts, similar to the other settlements that in this period characterized the Messapia [1]. Certainly, Aletion/Aletium was a settlement of some importance as early as the 6th century BC, but we do not know if it was affected in this period by urban development like other Messapian settlements, such as Cavallino and Oria. According to the distribution of findings from the Archaic and Classical periods, probably the Raggi area was at least partly peripheral to the settlement. It perhaps was concentrated on the hill of Lizza and could occupy a surface estimated between 14 and 24 hectares, but it cannot be certainly defined. It was characterized by nuclei of houses with stone foundations alternating with free spaces, partly also used as funerary areas. Several necropolises lay around the inhabited area; the main one of these was located north of the settlement, in the Tafuri area, today completely urbanized. Already at this epoch, the necropolis which extended on the north-western side of Monte D’Elia (65 m a.s.l.), located about 300 m south of the town, was used.

The available data for the classical period are very few and insufficient to establish how much the settlement of Aletion/Aletium was affected by the general transformation that during the 5th century BC affected various other Messapian centres, such as Oria and Ugento [5]. However, during the 5th century BC, the necropolis of Monte D’Elia shows a continuity of use. Most of the archaeological data about Aletion/Aletium is dated between the second half of the 4th and the first half of the 3rd century BC [5]. It was hypothesized that in this period the settlement was defended by city walls [4,8], of which no certain remains are preserved. On the basis of traces visible in the aerial photographs of the 1940s, a perimeter of 3.400 m was hypothetically reconstructed for these walls, enclosing a surface of about 67 hectares and including both the hill of Lizza and the Raggi area [1]. In the lack of data on housing, the indirect evidence to the flowering of the settlement in this phase comes almost exclusively from the necropolises that surrounded it and among which the necropolis of Monte D’Elia stands out, as in previous phases.

The ancient settlement of Alezio seems to have maintained a certain importance even after the Roman conquest of Salento, marked by the triumph over the Calabri and Sallentini in 266 BC. According to the available archaeological evidence, in this phase the settlement seems to be decreasing, concentrating on the hill of Lizza and in the central-western sector of the Raggi area. Between the late Republican age and the Imperial period, the settlement seems to lose a large part of its urban character to undergo a strong process of ruralization, marked by the presence of facilities for the transformation of agricultural products, such as olive oil and wine. The settlement survived until the Middle Ages with a progressive abandonment of housing structures and depopulation.

## 3. Topographical and Archaeological Survey

### 3.1. Topographical and 3D Survey

The topographical survey and the graphical representation of the Messapian necropolis of Monte d’Elia contributed to verify and to correct the survey made during the archaeological excavations in the 1980s. It has allowed us to update the cartography for a critical reading of the site: a useful tool for the investigation of the subsoil introduced in this paper.

Different indirect survey techniques have been used since the use of a single type of measurement does not give good results in terms of geometric accuracy, portability, automatism and photorealism. The sensors used for the survey use light radiation; a further distinction can be made depending on the nature of the light used to make the measurement. If natural light is used, measuring instruments are defined as “passive” (photogrammetric technique, Image-Based, etc.); if the light is generated in the measurement process, we are talking about “active sensors” (laser scanners, radars, total stations, etc.). The integration between different systems of three-dimensional relief represents a central topic of research in different disciplinary fields, within which new methods are being studied to integrate various techniques in an automatic or semi-automatic manner, enhancing their potential [9]. Both active and passive sensors show a high level of complementarity.

Digital photogrammetry is very useful when there is a limited permanence in the place of the surveyors or if the artefact can be described exhaustively by control points and lines. On the contrary, laser scanner requires more time in data collecting and in the post-processing phase from merging of the cloud points up to the processing of the polygonal mesh.This technique clearly offers very precise data not achievable with traditional photogrammetry. The use of different surveying instruments offers metric data characterized by multiresolution and allows to achieve an exhaustive result with the different representation of all geometries [10].

**Figure 2:**
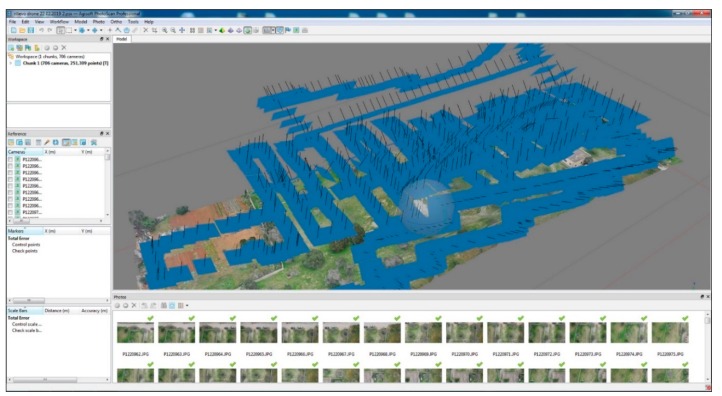
The 3D survey of the archaeological site of Monte d’Elia created with the Agisoft Photoscan software.

The archaeological park of Monte D’Elia has a total area of about 11,000 square meters. Inside it, only a part of the necropolis (corresponding to a surface of about 636 square meters) is currently visible. For a fast survey of the entire park, aerial digital photogrammetry was performed using an Unmanned Aerial Vehicle (UAV), while for the necropolis area, unfortunately covered by a metal roof, the survey using close range digital photogrammetry has been implemented with e laser scanner data (Figure 2).

The UAV airframe used was a Scrabble 4HSE quadcopter designed and manufactured by Italdron (Ravenna, Italy) and equipped with a high resolution digital camera (DMC-GH4, 16 Mpx, focal length: 28.0 mm; sensor resolution. 4608 × 2592 pixels, Panasonic, (Milano, Italy), fixed on a 3-axis gimbal. The maximum weight was 12 kg, allowing a maximum flight endurance limit of 10 min. To cover the whole area, flights were performed with a nadir orientation of the camera. Three additional flights with manual piloting with a 45° camera were carried out to collect oblique aerial images. The redundant set of images acquired at the selected timing facilitates the SfM approach. In total, 706 aerial photos have been taken using a constant shooting distance of about 5 m, at a midrange distance from the ground of 30 m.

The entire archaeological park was covered with a sufficient image overlap (about 70–80%), essential in order to get the tracking points in space and their resulting 3D position. The processing of the images by Agisoft Photoscan software (v. 1.4.0.5076, Agisoft, St. Petersburg, Russia) led to the alignment of the photos with a minimum margin of error (0.6/2.0 pixel) and to the creation of a dense cloud of about 19 million points. The model obtained was georeferenced through four ground control points (GCPs) located on the corners of the park and collected with a total station, to guarantee its correct scaling and orientation [11,12,13,14].

**Figure 3:**
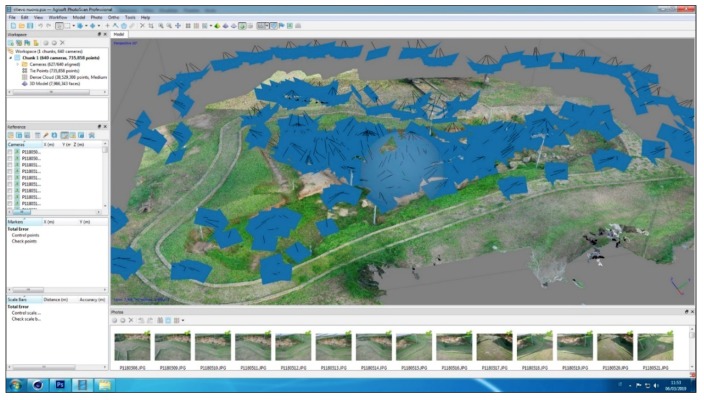
The 3D survey of the tombs currently visible at necropolis of Monte D’Elia created with the Agisoft Photoscan software.

**Figure 4:**
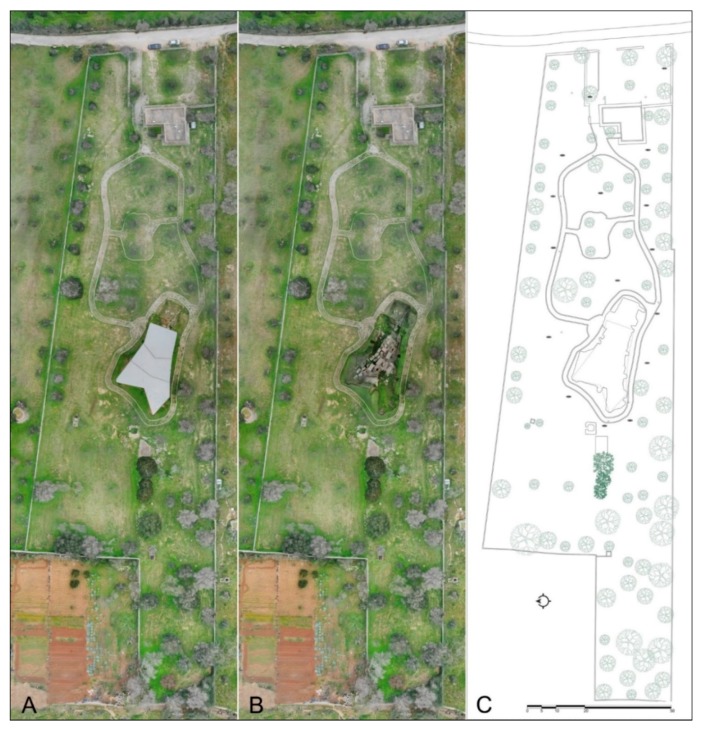
The 3D survey: (**A**), the digital aerial photogrammetry of the archaeological site; (**B**), the aerial survey merged with the digital close range photogrammetry of the tombs current visible under the metal cover; (**C**), CAD plan.

In the same Photoscan project a second “chunk” was detected in which the images of the second photographic survey of the area not covered from the UAV (due to the metal roof protecting the visible tombs) were aligned. Still with the same camera and by means of a telescopic pole, 627 photos were taken. The alignment of all photos has a margin of error 0.3/1.4 pixel and produced a final digital model of 8 million polygons with a texture of 15,000 × 15,000 px resolution (Figure 3). Both “chunks” were merged by placing markers in the common points, thus obtaining a single 3D model used as a basis for placing geophysical prospecting results (Figure 4).

**Figure 5:**
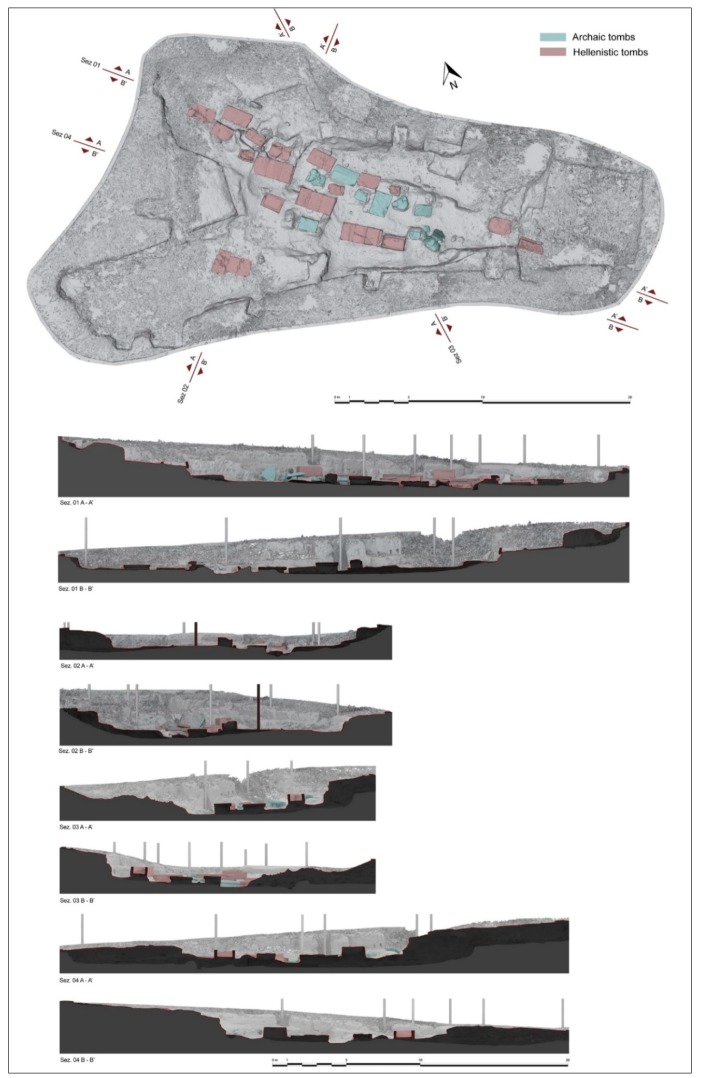
Laser scanner survey of the archaeological site of Monte D’Elia: plan and sections.

After analysing the morphological characteristics of the site and the presence of several sarcophagi and chests tombs, we have set up scanning stations at an average distance of about 5 to 7 m, between each other, in order to have a good coverage of all surfaces. The survey was performed with a P20 laser scanner (Leica, Milano, Italy) using an accuracy of 6 mm on a dome of 10 m radius [15,16,17,18]. The matching of point clouds was done manually. From them, a three-dimensional model was obtained with a mesh of about 7 million polygons with a resolution of 10 mm. Plan and sections of the area have been processed (Figure 5) starting from the scaled model.

Finally, the work flow planned and the data obtained allowed us to manage all information within a single 3D work space, and to produce two digital models aimed at a quickly providing all data about the geometry and the colour of the analysed scenario.

The integration between these techniques demonstrates how single instruments are characterized by a level of complementarity that makes an integrated system more powerful and flexible, able to provide a much better result in absolute terms and able itself to adapt to the single morphological features of the different objects contained in the detected scene. The complementarity of the techniques optimize the acquisition and modelling process, exploiting the maximum potential of the single instrument. The result achieved is a digital cognitive model, which is a repository of information; therefore, search queries can be carried out at various levels (Figure 4B).

### 3.2. Archaeological Map

**Figure 6:**
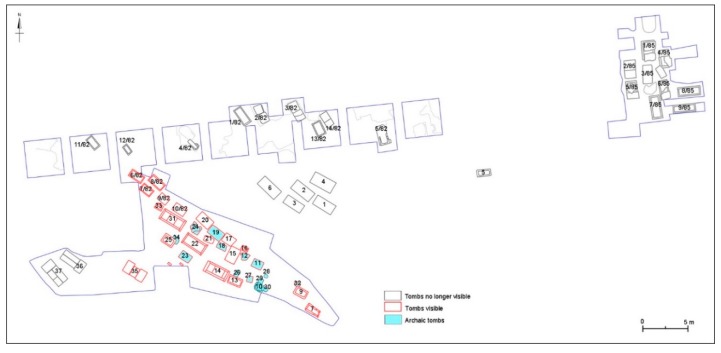
Plan of the excavated area of Monte D’Elia necropolis.

Between 1981 and 1985, archaeological excavations were carried out in the Messapian necropolis of Monte D’Elia. The investigations involved a large sector of the necropolis and they brought to light about 60 tombs, whose grave goods attest that the use of the funerary area, related to the settlement of Aletion/Aletium, was between 6th and 2nd century BC (Figure 6) [1,3,4,5,19,20,21,22,23,24,25,26]. The funerary types vary from the pit graves with cover consisting of a slab of local calcarenite, dating in the Archaic period, to the monolithic sarcophagi and to the large chests made of more calcarenite slabs, covered by one or more slabs, and in use between 5th and 3rd century BC. Various not inscribed cippi in calcarenite have also been found, associated with both Archaic and Hellenistic tombs [24,27,28]. They are of parallelepiped shape (exemplifying measures: 34 × 22 cm, height 65 cm).

The discovered tombs were buried in 1989 for conservation reasons and largely brought to light again in 2004, when the area became an archaeological park. The available documentation is incomplete, the published works are few and the plans of the excavated areas are inconsistent with each other and without georeferencing. In particular, only two general plans are available: the first, drawn by Duma and Zingariello, and preserved in the archive of the Superintendent for the Archaeological Heritage of Puglia Region, documents the excavations of 1981–1982; the second one, drawn by Danese in 1987, shows both the excavations of 1981–1982 (but with some differences compared to the Duma-Zingariello map) and those of 1985 (this last survey is kept in the archive of the Municipality of Alezio and was published in 1981 [3] (Figure 2) and [5] (Figure 5) and, then, in [1] (Figure 4, with changes). The currently visible area of the necropolis includes most of the tombs brought to light in 1981 and three of those excavated in 1982. The execution of a new plan of the visible tombs, georeferenced in the new topographical map of the site, allowed evaluation of the accuracy of the reliefs of Duma-Zingariello and Danese (which in previous publications was always oriented to the south). In particular, the Duma-Zingariello drawing (which also shows the official denomination and numbering of the graves unearthed in 1981 and part of those discovered in 1982) was very faithful to the real situation and therefore considered reliable even for the tombs no longer in sight. The plan of Danese, on the other hand, was less precise, but it is the only documentation available for the tombs unearthed in 1985. Furthermore, the comparison of the plan of Duma-Zingariello with the current situation showed that at the time of the reopening of the excavations, in 2004, some of the Archaic tombs brought to light in 1981, which had more fragile structures, were destroyed, while others were slightly moved from their original position. Moreover, none of the plans show the heights above ground level or sea level are never shown.

The production of a new archaeological map of the necropolis, including all the discovered tombs, and the contextualization of data from grave goods and epigraphic documentation allowed some considerations and observations about the funerary area of Monte D’Elia and its organization. The excavation of 1981 is undoubtedly the best documented, thanks also to a preliminary study of the grave goods that allowed establishing the general chronology of the necropolis. This excavation involved an area extending 35 m in the east-west direction and 17 m in the north-south direction and led to the discovery of 37 tombs. The tombs referring to the Archaic period according to typology and grave goods, and datable between the 6th and the beginning of the 5th century BC, are at least ten (nos. 8, 10, 11, 12, 18, 19, 23, 24, 26, 29); we can also probably add the tombs nos. 28, 32, 34, characterized by the same typology. With the exception of the tomb no. 19, which is a sarcophagus (1 × 1.65 m) and is dated between the end of the 6th and the beginning of the 5th century BC, the others tombs are all pit graves or excavated in the calcareous bench (exemplifying measures: 60 × 80 cm) and covered by a calcarenite slab (max size 0.85 × 1.25 m). The deceased appear in a crouched position and the grave goods include a small number of objects [1,3,19,24,25,29]. They generally comprising a small olla and a one handle jug, sometimes accompanied by a one handle uncoloured cup, all of local production, for female burials (nos. 12, 18, 19), and a krater associated with an imported drinking cup (Ionic cups type B2 or imitations of Attic cups Bloesch C) and other vessels of local production (such as jugs or small jugs ladle, lekanai, and stamnoi), for the male tombs (nos. 8, 10, 11, 23, 24, 26, 29). In particular, the more ancient male burials (nos. 10, 11, 24, 29), datable to the second half of the 6th century BC, are characterized by the presence of globular Messapian kraters with mushroom-shaped handles accompanied by Ionic cups of type B2, likely produced at Metaponto. The more recent tombs (nos. 23 and 26), dating between the end of the 6th and the beginning of the 5th century BC, are characterized instead by the presence of a column-krater that constitutes a local imitation of Greek models and is always associated with imitations of the Attic cups like Bloesch C, referring both to the production of Taranto that to the Corinthian colonies of the Adriatic Sea [29]. A few metallic materials were found, mostly related to fibulae.

The new relief of the still visible tombs has highlighted that the nos. 10, 26, 28, 29, 30 and 34, documented in the Duma-Zingariello plan and in some photos collected in 1981 [3] (Figure 3) and [5] (Figure 6), were unfortunately destroyed during the excavation of 2004, while the remains of the nos. 12 and 24 have been slightly displaced with respect to the original position. The location of tomb no. 8, today not preserved and absent in the Duma-Zingariello plan, is uncertain.

The most consistent group of tombs dated between the 5th and 3rd century BC and it consists of sarcophagi (nos. 5, 9, 13, 15, 16, 17, 21, 25, 33) and chests made of slabs (nos. 1, 2, 3, 4, 6, 7, 14, 20, 22, 31, 35, 36, 37). Among the first group, sarcophagus no. 5 (m 1.65 × 0.65, height m 0.45) was found to the east of the investigated area, close to a cippus, and was transferred in the garden of the Museo Civico Messapico of Alezio. Other three tombs (nos. 16, 21, 33) are very small (for example 50 × 80 cm). Among chests of slabs (exemplifying measures: lengths 1.74–2.97 m, widths 0.86–1.33 m, heights 0.70–1.20 m), often characterized by cover slabs provided with recesses for the handle, eight (nos. 1, 2, 3, 4, 6, 20, 22, 31) have Messapian inscriptions engraved on the inner faces of one side slab and bearing the names of the owners of the tombs themselves. They are datable between the second half of the 5th and the end of the 3rd-beginnings of the 2nd century BC [1,7,30,31,32,33]. About this group of tombs, four (nos. 1, 2, 3, 6) were transferred in the garden of the Museo Civico Messapico of Alezio and belonged to a nucleus located in the north-eastern sector of the investigated area, including also tomb no. 4, today buried.

The grave goods of these tombs have not yet been carefully studied (see notes in [1,4,5,25]). In some cases, they go back to the 2nd century BC (for example the tombs nos. 14 and 31) suggesting their reuse or use over several generations. The grave goods generally include local trozzelle and lekanai of local production, black-painted pottery (such as lekythoi, patere, skyphoi, oil lamps), various types of uncoloured vases, balsamari, as well as small female statues sitting on a throne or standing (as in the tombs nos. 3, 6, 22), egg shells (as in the tombs nos. 3, 6, 15) and few metallic objects (such as an iron brooch and a bronze earring from tomb no. 3).

The excavation carried out in 1982 involved an area of almost 50 m wide in the east-west direction, located just north of that investigated in 1981. Regular squares of about 4 × 4 m were excavated, and in some cases were further enlarged. At least 11 tombs (sarcophagi and chests made of slabs) were found (nos. 1/82–11/82); three others tombs may be added (nos. 12/82–14/82), which are drawn in the Danese plan but not in Duma-Zingariello plan. Only sarcophagi nos. 6/82–10/82 are currently visible and they are located at the north-western end of the tombs brought to light in 1981. The rest is buried, as well as all the tombs (at least nine), always sarcophagi and chest of slabs, brought to light in 1985 to the east of the area investigated in 1982, where an excavation of about 11 × 8 m was carried out. Some slabs of the north-eastern excavated sector (tombs nos. 1/85–9/85) were removed (some of these slabs are piled in the vicinity), but we don’t have further data about the tombs that are still in situ and buried. It is possible that also in the northern excavated sector (nos. 1/82–12/82) some tombs were removed.

Unlike the excavation of 1982 which did not allow the recovery of new inscriptions, three chest tombs discovered in 1985 (example measurements: lengths 1.25–2.23 m, widths 0.80–0.88, heights 0.60) returned three Messapian epigraphs of the 4th–3rd century BC [1,7,34]. The inscriptions, while awaiting the study of the grave goods, allow us to date at least part of these tombs to the Late-Classical and Hellenistic age. It should be emphasized that there are no data about the presence of Archaic tombs, even in the areas investigated in 1982 and 1985.

**Figure 7:**
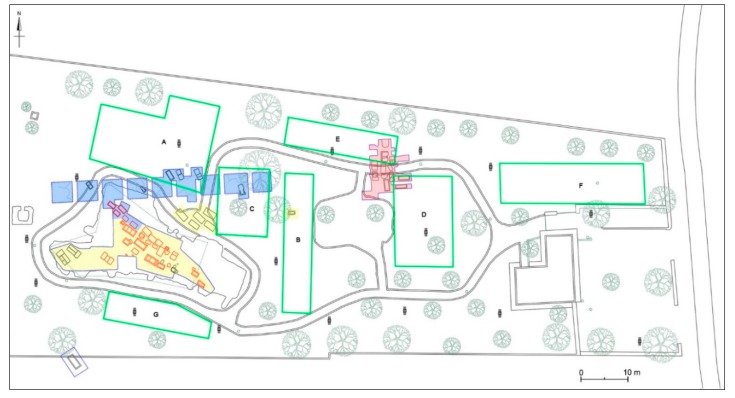
The new map of the funerary area of Monte D’Elia on which the seven investigated areas are highlighted.

Overall, the graves unearthed between 1981 and 1985 occupy an area extending 25 m in north-south direction and about 75 m in east-west direction (Figure 7). An isolated tomb was also brought to light approximately 25 m south-west of the area excavated in 1981. The necropolis was organised by terracing a natural slope slightly downhill towards the west, with the graves were positioned on different levels.

In the westernmost sector, characterized by a very thick layer of earth above the rocky bank, thanks to the still visible graves it is possible to see that the tombs were found starting from a depth of 70–80 cm and that the deepest ones lie at −1.5/−2 m compared to the ground level. In the western sector the tombs are all oriented in a north-west/south-east direction. It is possible that this orientation was influenced by an ancient road paved with compacted small stones, identified, only in a short section, in 2004 about 10 m north-west of the funerary area. The road, which perhaps ran between the main nucleus of the tombs brought to light in 1981 and the group consisting of tombs nos. 1–4 and 6, could be related to the north-west/south-east oriented road documented in 1999 by geophysical surveys, still unpublished, carried out by the University of Sydney [6,25]. Lastly, as we proceed towards the east, the tombs take on a marked north-south or east-west orientation, as is evident especially in the eastern sector excavated in 1985. The orientation of this area could be related to a different road network: indeed, it may be interesting to remember that the modern road bounding the eastern side of the archaeological park is identified with the survival of the so-called Via Sallentina [35], the main road that connected the ancient settlements of Aletium and Uxentum (today Ugento).

## 4. Geophysical Survey

A wide range of geophysical methods are applied in archaeology for obtaining high-resolution images of the subsurface. The geophysical method used in this study is based on the detection of variations in the electromagnetic properties of the subsoil and the use of these data to identify artefacts and distinguish between these and natural variations in the soil. Seven areas, labelled respectively A, B, C, D, E, F, and G were considered for GPR survey (Figure 7). It was necessary to choose seven different areas due to the current conditions of the archaeological park: in fact, the presence of numerous obstacles, such as walls, roads, trees, and light poles, did not allow us to cover the overall surface of the site.

### 4.1. GPR Data Acquisition and Analysis

The GPR survey was carried out with an IDS Ris Hi Mod system (IDS, Pisa, Italy) using a 200–600 MHz dual band antenna. Data were acquired in continuous mode on the overall surface of the seven investigated areas, along 0.5 m-spaced survey lines, using 512 samples per trace, 80 ns time window for 600 MHz antenna and 160 ns time window for 200 MHz antenna, and a manual time-varying gain function. Transect spacing should be less than one half the wavelength of possible reflections returned from the smallest target to be mapped [36,37,38].

The data were subsequently processed using standard two-dimensional processing techniques by means of the GPR-Slice Version 7.0 software [39]. The processing flow-chart consists of the following steps: (i) header editing for inserting the geometrical information; (ii) frequency filtering; (iii) manual gain, to adjust the acquisition gain function and enhance the visibility of deeper anomalies; (iv) customized background removal to attenuate the horizontal banding in the deeper part of the sections (ringing), performed by subtracting in different time ranges a ‘local’ average noise trace estimated from suitably selected time-distance windows with low signal content (this local subtraction procedure was necessary to avoid artefacts created by the classic subtraction of a ‘global’ average trace estimated from the entire section, due to the presence of zones with a very strong signal); (v) estimation of the average electromagnetic wave velocity by hyperbola fitting; (vi) Kirchhoff migration, using a constant average velocity value of 0.098 m/ns. The migrated data were subsequently merged together into three-dimensional volumes and visualized in various ways in order to enhance the spatial correlations of anomalies of interest.

**Figure 8:**
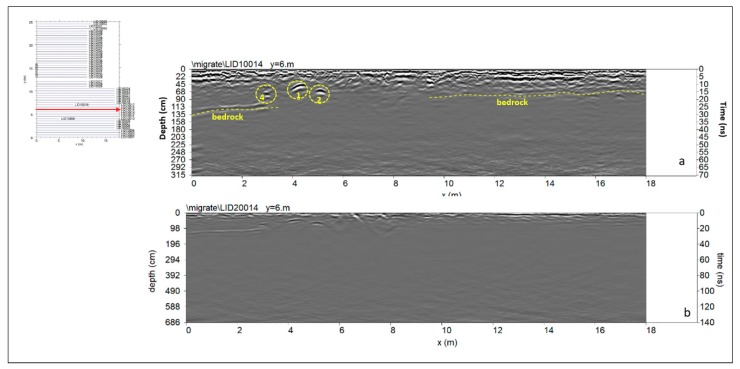
Area A: (**a**) 600 MHz antenna: reflection events related probably to a modern pipe (4), tombs (1, 2) and to the bedrock, at a depth between 0.75 to 1.35 m; (**b**) 200 MHz antenna: no more evidenced were found below 130 ns.

A way of obtaining visually useful maps for understanding the plan distribution of reflection amplitudes within specific time intervals is the creation of horizontal time slices. These are maps on which the reflection amplitudes have been projected at a specified time (or depth), with a selected time interval [40]. In a graphic method developed by Goodman et al. [41], termed ‘overlay analysis’, the strongest and weakest reflectors at the depth of each slice are assigned specific colours. This technique allows the linkage of structures buried at different depths. This represents an improvement in imaging because subtle features that are indistinguishable on radargrams can be seen and interpreted in a more easily. In the present work the time-slice technique has been used to display the amplitude variations within consecutive time windows of width Δt = 5 ns. Moreover, the highest amplitudes were rendered into an iso-surface [36,42,43,44]. Three-dimensional amplitude iso-surface rendering displays amplitudes of equal value in the GPR study volume. Shading is usually used to illuminate these surfaces, giving the appearance of real archaeological structures. In this case the threshold calibration is a very delicate task in order to obtain useful results. In order to define the depth of archaeological remains the electromagnetic (EM) wave velocity, using the characteristic hyperbolic shape of a reflection from a point source (diffraction hyperbola), was used. Only the 600 MHz antenna results were considered due to the fact that no additional information were visible on the 200 MHz antenna results (see Figure 8b).

### 4.2. Geophysical Anomalies

#### 4.2.1. Area A

Area A was about 26 × 19 m. In this area an electromagnetic energy penetration depth of 40–50 ns was found. Figure 8 shows the processed radargram related to the 14th profile and to the 600 MHz antenna. It shows several hyperbolic shaped reflection events. The first reflection event, labelled 4, at two-way travel time window between 15 and 20 ns is clearly visible. Its size is about 0.2 m and the depth is between 0.68 and 0.90 m (with an average electromagnetic wave velocity of 0.09 m/ns). This could be related to a modern pipe. On each of the GPR records the lowest (dashed yellow) continuous and slightly undulating reflector appears strong and irregular and reaches a maximum depth below the ground surface ranging from 0.75 to 1.35 m. This event could be related to the shallow bedrock visible also in the old excavations (Figure 6). The reflection events labelled 1 and 2 are visible at depth from about 0.45 m to 0.60 m. The size (about 0.80 m) and shape suggest that they are probably related to the presence of filled tombs (Figure 8).

**Figure 9:**
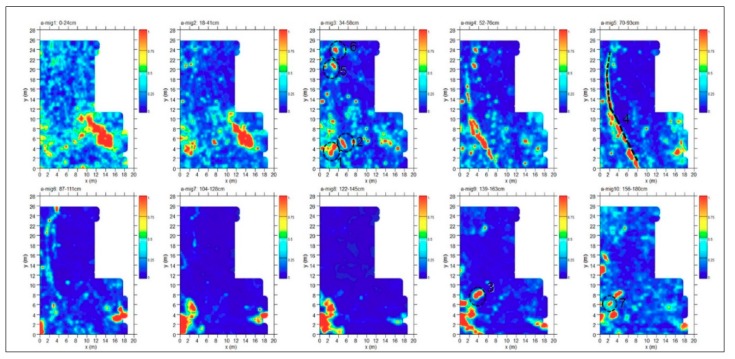
Time slices of Area A: the most significant anomalies, probably related to tombs, are highlighted at depth of 34–58 cm (1, 2, 5, 6) and 139–180 cm (3, 7). A modern pipe is at 70–93 cm (4).

In order to identify the depth evolution of buried structures, including their size, shape and location, time slices using the overlay analysis [41,45] were built (Figure 9). The time slices show the normalized amplitude using a range defined by blue as zero and red as 1. In the slices ranging from 0.34 m to 0.58 m depth, relatively high-amplitude alignments (labelled 1, 2, 5 and 6 respectively) are clearly visible. These correspond to the anomalies labelled 1 and 2 in the radargram (Figure 8). In the time slices (Figure 9) ranging from 0.70 m to 0.90 m depth the dashed dark line highlights a high-amplitude anomaly (labelled 4); in the same area, the latest two time slices ranging from 1.39m to 1.8m depth show other high amplitude events (labelled 3 and 7).

#### 4.2.2. Area B

**Figure 10:**
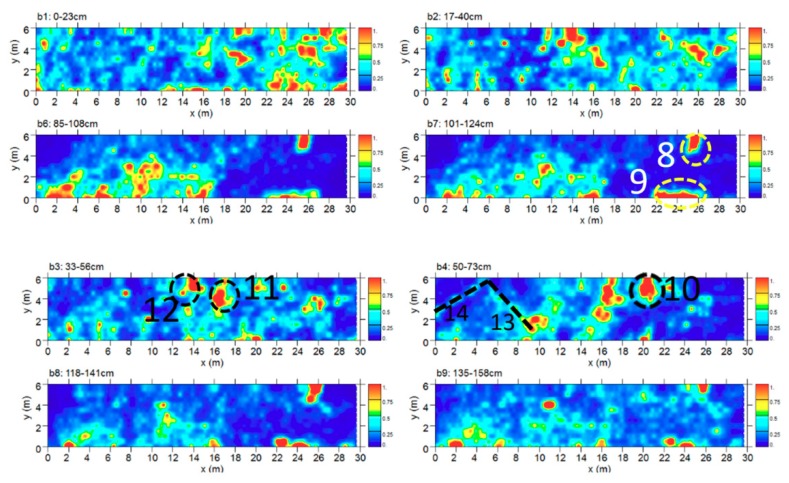
Time slices of Area B: the most significant anomalies, probably related to tombs, are highlighted at depth of 33–73 cm (10, 11, 12) and 101–124 cm (8, 9). A modern pipe is at 70–93 cm (4). Linear anomalies (13, 14) are visible at 50–73 cm (square structure?).

Area B was about 6 × 30 m in size. The time slice analysis (Figure 10) show several high electromagnetic energy amplitude events. In the slices ranging from 0.33 m to 0.56 m depth, relatively high-amplitude alignments (labelled 12, and 11, respectively) are clearly visible. These by their shape (rectangular) and dimensions (about 0.8 m × 1.8 m) could be related to the presence of tombs. In the time slices ranging from 0.50 m to 0.70 m depth the dashed dark line highlights a high-amplitude anomaly (labelled 13 and 14) that seems to indicate the presence of archaeological features (walls); in the same slice the anomaly labelled 10 is also visible. In the same area, deeper time slice ranging from 1.01 m to 1.24 m depth shows other high amplitude events (labelled 8 and 9). Also in this case the alignment with the other excavated tombs allows one to interpret these as probable tombs.

#### 4.2.3. Area C

**Figure 11:**
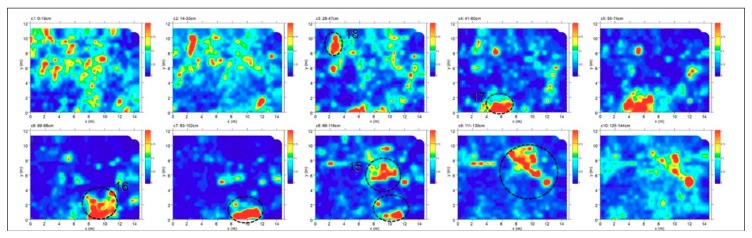
Time slices of Area C: the most significant anomalies, probably related to tombs, are highlighted at depth of 28–60 cm (17, 18) and 69–130 cm (15, 16).

Area C was about 15 × 13 m. The time slice analysis (Figure 11) show several high electromagnetic energy amplitude events labelled 15, 16, 17 and 18, respectively. The shape, the dimensions and the alignment with the other excavated tombs allow one to interpret these as probable tombs.

#### 4.2.4. Area D

**Figure 12:**
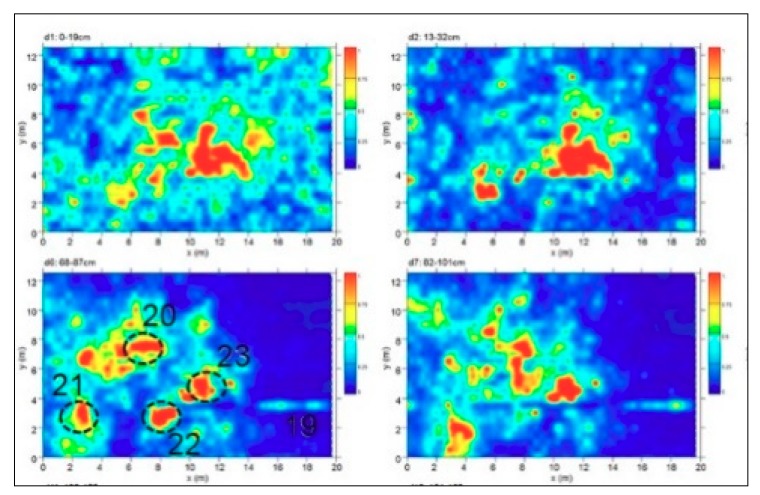
Time slices of Area D: the most significant anomalies, probably related to tombs, are highlighted at depth of 28–74 cm (20, 21, 22, 23). A linear anomaly (19) could be related to a buried structure or to the boundary of old excavation.

Area D was about 13 × 20 m. The time slice analysis (Figure 12) show several high electromagnetic energy amplitude events labelled 20, 21, 22 and 23, respectively. The shape, the dimensions and the alignment with the other excavated tombs allow one to interpret these as probable tombs.

#### 4.2.5. Area E

**Figure 13:**
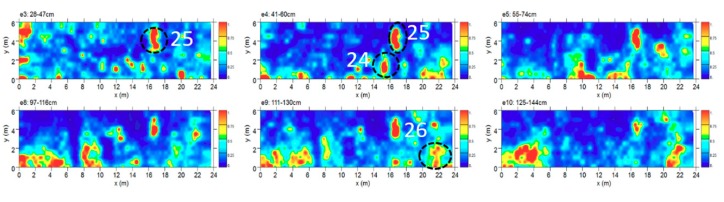
Time slices of Area E: the most significant anomalies, probably related to tombs, are highlighted at depth of 68–87 cm (24, 25) and 111–130 cm (26).

Area E was about 24 × 6 m. The time slice analysis (Figure 13) show several high electromagnetic energy amplitude events labelled 24, 25 and 26, respectively. The shape, the dimensions and the alignment with the other excavated tombs allow one to interpret these as probable tombs.

#### 4.2.6. Area F

**Figure 14:**
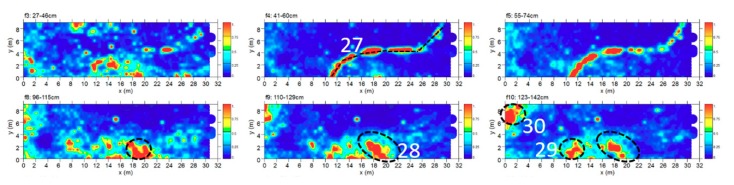
Time slices of Area F: the most significant anomalies, probably related to tombs, are highlighted at depth of 96–142 cm (28, 29, 30). An evident and wavy anomaly at 41–74 cm (27) is identifiable as a modern pipe.

Area F was about 31.5 × 9 m. The time slice analysis (Figure 14) show several high electromagnetic energy amplitude events labelled 27, 28, 29 and 30, respectively. The shape, the dimensions and the alignment with the other excavated tombs allows these to be interpreted as probable tombs. The anomaly labelled 27 was attributed to a modern pipeline.

#### 4.2.7. Area G

**Figure 15:**
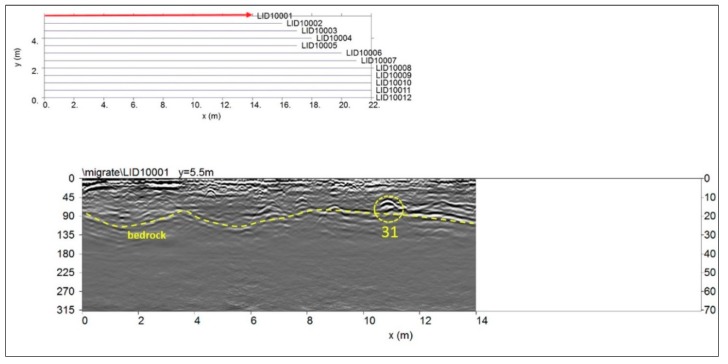
Area G: reflection events probably related to a tomb (31), and to the bedrock, at a depth between 0.8 to 1.30 m.

Area G was about 23 × 6 m. In this area an electromagnetic energy penetration depth of 40–45 ns was found. Figure 15 shows the processed radargram related to the 1st profile and to the 600 MHz antenna. It shows several hyperbolic shaped reflection events. The first reflection event (dashed yellow) continuous and slightly undulating reflector appears strong and irregular and reaches a maximum depth below the ground surface ranging from 0.8 to 1.30 m. This event could be related to the shallow bedrock visible also in the old excavations (Figure 6). The reflection events labelled 31 is visible at depth from about 0.40 m to 0.60 m. The dimension (about 0.80 m) and shape suggest that it is probably related to the presence of a tomb. Particularly it is important to underline that this event show a polarity inversion of the electromagnetic wave and this is related to the presence of void [38].

**Figure 16:**
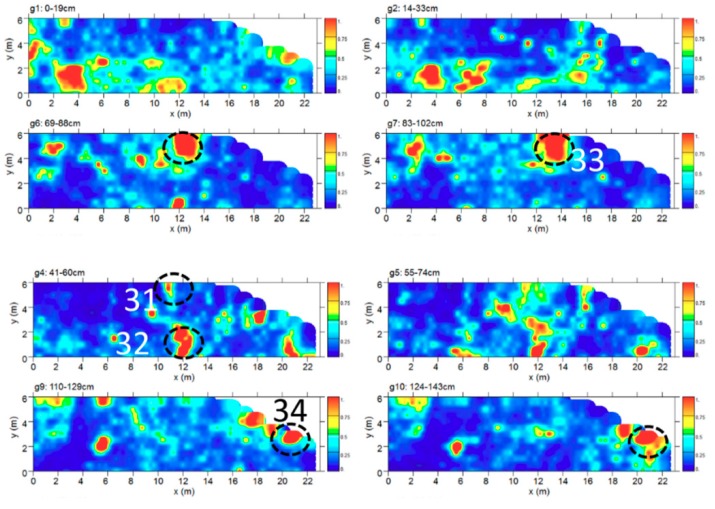
Time slices of Area G: the most significant anomalies, probably related to tombs, are highlighted at depth of 41–60 cm (31, 32), 69–102 (33) and 110–143 (34).

The time slice analysis (Figure 16) show several high electromagnetic energy amplitude events labelled 31, 32, 33 and 34, respectively. The shape, the dimensions and the alignment with the other excavated tombs allow these to be interpreted as probable tombs.

### 4.3. Integration of Topographical and Geophysical 3D Data

**Figure 17:**
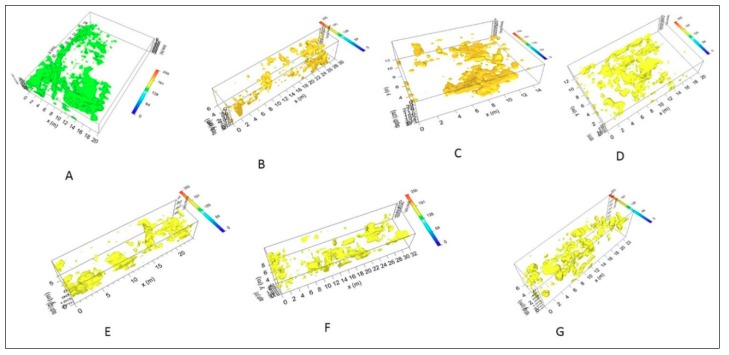
Areas A-G: the collected data displayed set with iso-amplitude surfaces, using a certain percentage of the maximum complex trace amplitude threshold value.

The integrated analysis between the 3D topographical and geophysical data allowed for a better understanding of the nature of the detected GPR anomalies. In some cases, the archaeological interpretation of GPR measurements was favoured by displaying the acquired data set with iso-amplitude surfaces, using a certain percentage of the maximum complex trace amplitude threshold value (Figure 17). Obviously, lowering the threshold value increases the visibility of the main anomaly and smaller objects, but also the heterogeneity noise. Relatively strong continuous reflections are visible on the threshold volumes. This visualization technique portrayed better the evidence of the anomalies found in the surveyed areas.

**Figure 18:**
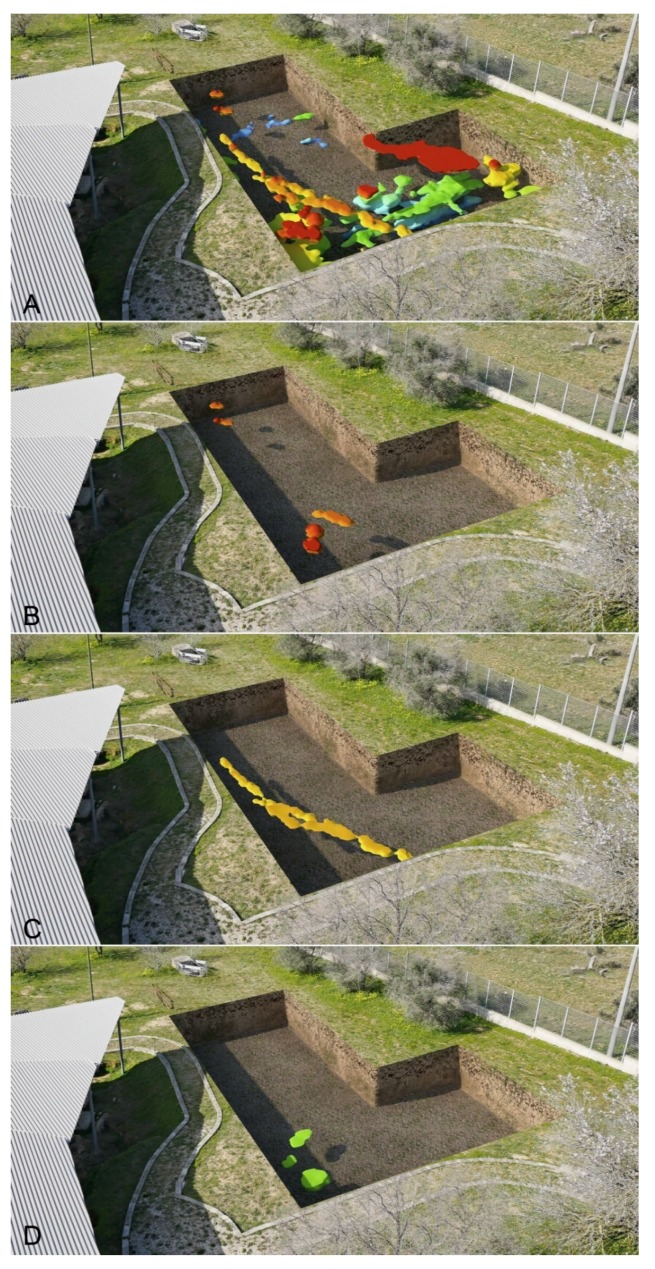
3D reproduction of the anomalies under the soil in the area A inserted in a digital photo: (**A**) general view of the anomalies; (**B**) slice of the anomalies at depth of about 60 cm; (**C**) slice of the anomalies at depth of about 90 cm; (**D**) slice of the anomalies at depth of about 180 cm.

In this work, an integration of 3D topographical information with the GPR data results was necessary first of all to understand the context and continuity of the structures found. It demonstrates that geophysical analysis can be very useful in order to place archaeological structures into a three-dimensional framework. Archaeological and topographical information merged with GPR mapping of the buried units leads directly to a better understanding of the topography of the necropolis (Figure 18) and facilitates the reading and therefore the interpretation of the spatial relations between the various anomalies at different depths.

**Figure 19:**
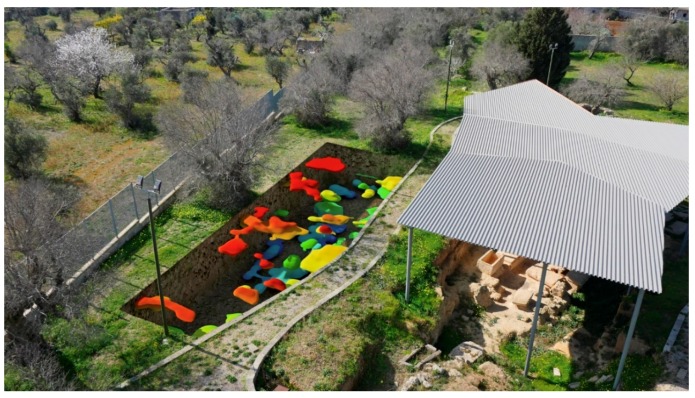
3D reproduction of the anomalies under the soil in the area G.

An important aspect of the research carried out was to identify those methodologies that could illustrate the results of geophysical surveys filling the communication gap that often makes them hard to understand. In this project the aid of computer graphics has been necessary to realize synthesis images calculated starting from three-dimensional environments. The first step was to create three-dimensional models from the georadar iso-surfaces volumes file: the meshes were then correctly oriented and scaled in relation to the size of the individual areas investigated. The polygonal surfaces have been mapped using a gradient texture that matches the altimetric map, on the axis of height, with a range of colours that from the most superficial areas (red colour), go with the different shades to indicate the different dimensions up to that deeper (blue colour). In this way, three-dimensional simulations have been carried out on the perspective views of the individual areas explored, making immediately comprehensible the data obtained, both as in the whole and in the specific levels. The most important aspect obtained is an overview that can simultaneously compare the uncovered funerary structures with those detected by the geometrical data: a relationship between the visible and the non-visible able to provide new information on the structures and the extension of the necropolis. This is useful for the development of the most up-to-date considerations of a topographical nature of the site and to offer further elements in the planning of next archaeological excavation (Figure 18 and Figure 19).

### 4.4. Archaeological Interpretation

The seven areas investigated by georadar prospecting are close to the sector of the funerary area excavated in 1981–1982 and still partially visible (see Section 3.2), lying to the north, south and east of it (Figure 7). In the eastern sector, some areas partially overlap the areas investigated during the archaeological campaigns of 1982 and 1985. Considering the maximum depth to which anomalies with archaeological interest are found (m 1.4–1.5 max.), the best results are offered by the 600 MHz antenna, which allows for a detailed reading at different depths. Moreover, these results are always confirmed by the 200 MHz antenna. The time slices of each area were georeferenced in the new topographical map of the necropolis, in order to define a precise location of the anomalies and to compare them with the already known archaeological features.

The surface of the investigated areas is flat and the land on which the necropolis extends is slightly sloping from east to west. Geophysical surveys have shown that the rocky bank is not very deep (generally between 0.8 and 1.4 m). It is less deep in the eastern portion of the archaeological park, while it tends to deepen towards north and north-west. Overall, in each investigated area, anomalies of archaeological interest were found, very useful for the study of the organisation and extension of the necropolis. The interpretation of mostly of these anomalies as tombs was facilitated by their georeferencing in the new archaeological map of the necropolis, which collect all the already known archaeological data. Indeed, the interpretation was mostly based on the geophysical characteristics of the anomalies and their sizes and orientation in relation to the already known tombs.

In particular, the georadar survey has highlighted about 25 anomalies that can be interpreted as further tombs (chests of slabs or sarcophagi), evidencing the extension of the necropolis beyond the limits known to date. Indeed, further possible tombs were found in a strip of land (Area A) located just north of the stratigraphic excavations carried out in 1982 and in the area located immediately south (Area G) of the large area investigated in 1981; at this regard, the extension of the necropolis in this direction was already suggested by the isolated tomb found about 25 m south of the 1981 excavation area, in correspondence of the enclosure wall of the archaeological park.

The results of the B and C Areas, partly overlapped on the sectors excavated in 1981–1982, now filled in, attest the presence of further tombs outside those sectors. In the same way, the results of the D and E Areas, partly overlapped on the sector excavated in 1985, also filled in, suggest an extension of the necropolis beyond the boundaries established in 1980s. The latter data seems to be also documented by the results of the georadar survey of Area F, where other anomalies were found, perhaps also related to tombs; these data are very interesting, since the archaeological excavations conducted in 2004 immediately further south, in correspondence with the building at the entrance of the Archaeological Park, had not found ancient evidence [25]. Obviously, only future stratigraphic excavations could confirm the identification of these anomalies with tombs, but surely the analisys of the results of the georadar surveys will be able to address future archaeological research. Moreover, stratigraphic excavations could allow to identify also further Archaic tombs consisting of small pit graves generally covered by a slab, which are more difficult to detect among the other anomalies highlighted by GPR prospecting in this area. The most significant anomalies of each area are examined below.

#### 4.4.1. Areas A-C

**Figure 20:**
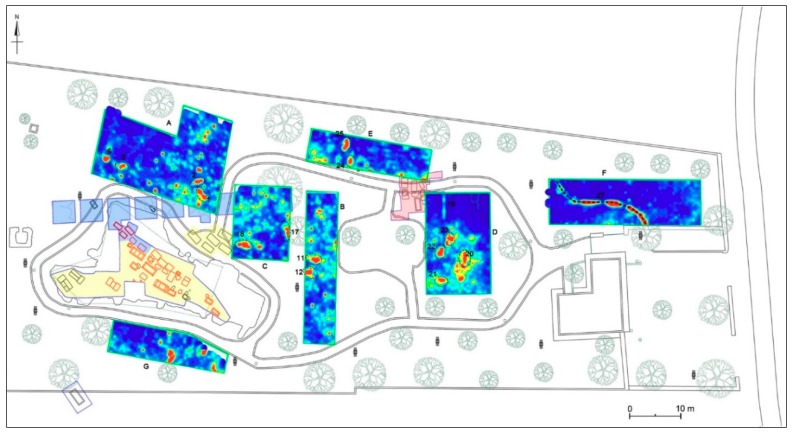
Archaeological map of the Monte D’Elia necropolis with the georeferenced time-slices of the seven areas investigated: A: time-slice at 34–58 cm depth; B: time-slice at 33–56 cm depth; C: time-slice at 28–47 cm depth; D: time-slice at 68–87 cm depth; E: time-slice at 41–60 cm depth; F: time-slice at 41–60 cm depth; G: time-slice at 41–60 cm depth.

Area A has an irregular shape of about 380 m^2^ and is located immediately north of the area investigated in 1982, overlapping partially its south-eastern end. At the depth of 34–58 cm some anomalies referable to filled cavities (nos. 1–2, 5–6) can be seen (Figure 20). They could be identified with tombs made of chests of slabs or sarcophagi due to their shape and size. In all cases, these anomalies do not seem deeper than 70–90 cm, a depth where the rocky bank already is attested in the southern part of Area A. The rocky bank is sloping towards north and north-west, where it is m 1.35 deep.

**Figure 21:**
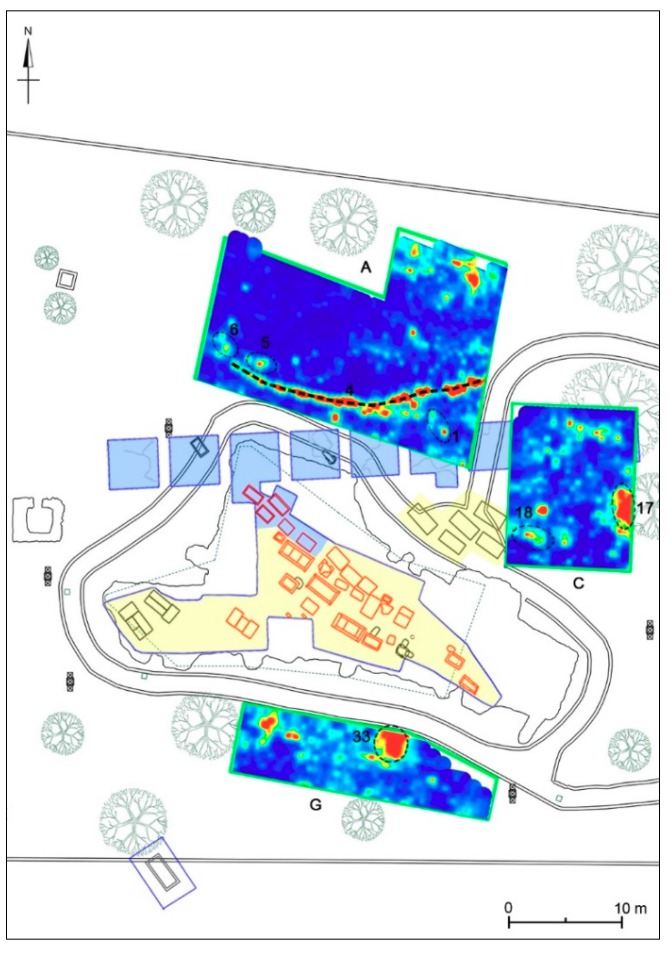
Eastern sector of the investigated area. A: time-slice at 70–93 cm depth; C: time-slice at 41–60 cm depth; G: time-slice at 83–102 cm depth.

**Figure 22:**
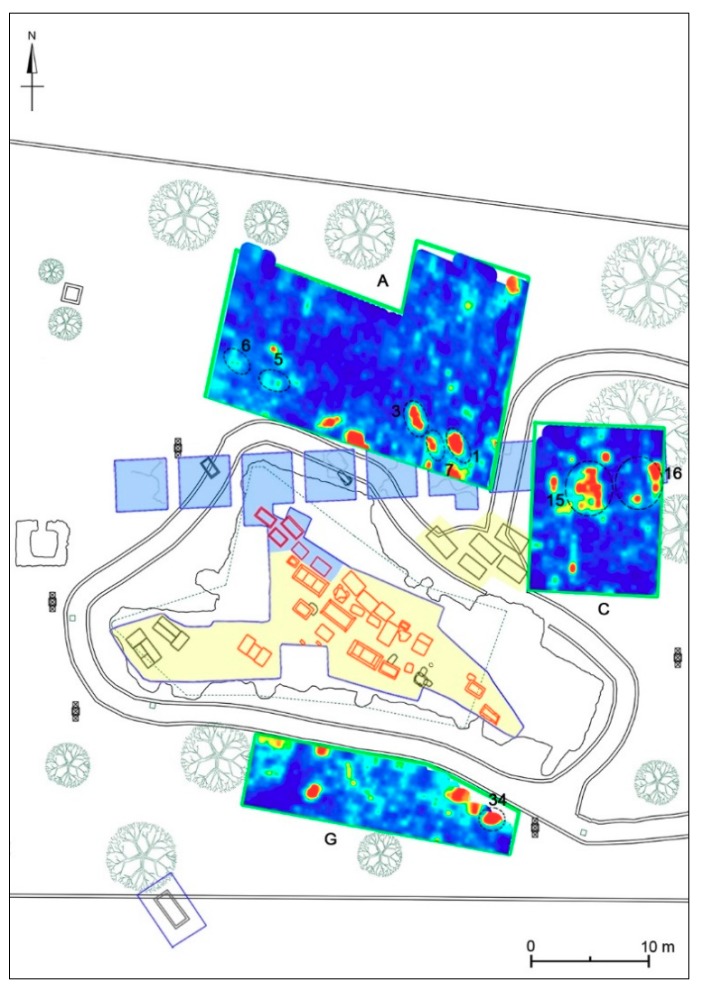
Eastern sector of the investigated area. A: time-slice at 156–180 cm depth; C: time-slice at 98–116 cm depth; G: time-slice at 110–129 cm depth.

The identification with tombs seems likely for anomalies nos. 5–6, confirming continuation to the north-west of the tombs brought to light in 1982. The most problematic interpretation concerns the anomalies nos. 1–2; the first falls in an area partially excavated in 1982 and may be due to less compact soil following the archaeological excavations, while the no. 2 has a divergent orientation from the nearby tombs. It is possible that it is in some connection with the evident anomaly no. 4, visible from about 50 to 100 cm deep, oriented roughly in the east-west direction and crossing the entire southern portion of the investigated area (Figure 21). This anomaly perhaps corresponds to a pipeline, probably modern, which seems to have been laid avoiding the structures that determined the anomalies nos. 5–6. There are some modern structures, now largely destroyed, in the north-western sector of the park and it is possible that the pipeline was directed to this area. Between the depths of 1.40 and 1.80 m, another anomaly (no. 3) can also be seen (Figure 22), referable to a cavity elongated in the north-west/south-east direction, which could be identified by shape, orientation and size with a chest of slabs placed very close to the tomb no. 2/82 brought to light in 1982 (see Section 3.2), arranged in a recess of the rocky bank and perhaps documented by another anomaly visible approximately at the same depth.

**Figure 23:**
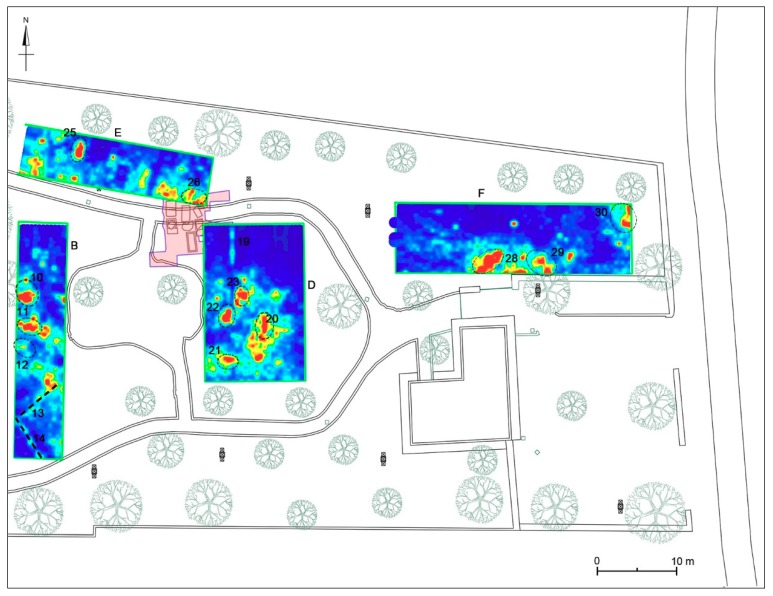
Western sector of the investigated area. B: time-slice at 50–73 cm depth; D: time-slice at 68–87 cm depth; E: time-slice at 111–130 cm depth; F: time-slice at 110–129 cm depth.

Area B has a rectangular shape of 6 × 30 m (180 m^2^) and is between the area excavated in 1981–1982 and the area investigated in 1985. The georadar measurements documented the bedrock already at a depth of 90–100 cm in the central and southern part of the area, while to the north it is instead found at −130/−140 cm. In the layer of soil above the bedrock, various anomalies with archaeological interest have been found. In particular, between 30 and 70 cm of depth we can see two anomalies (nos. 11–12) oriented roughly in the east-west direction (Figure 20 and Figure 23) and referable to cavities, which for orientation and dimension could be identified with tombs, made of stone slabs or sarcophagi, like the sarcophagus no. 5 unearthed in 1981 immediately further north; it was recovered a few centimeters of depth together with a cippus and then transported to the Museo Civico Messapico of Alezio (see Section 3.2). The anomaly no. 10 (Figure 23), visible up to a depth of 90 cm, could have been produced by remains of the excavation activities carried out to bring to light this sarcophagus: the high reflection could be due to the uncompact soil and stones used for filling the archaeological excavation.

**Figure 24:**
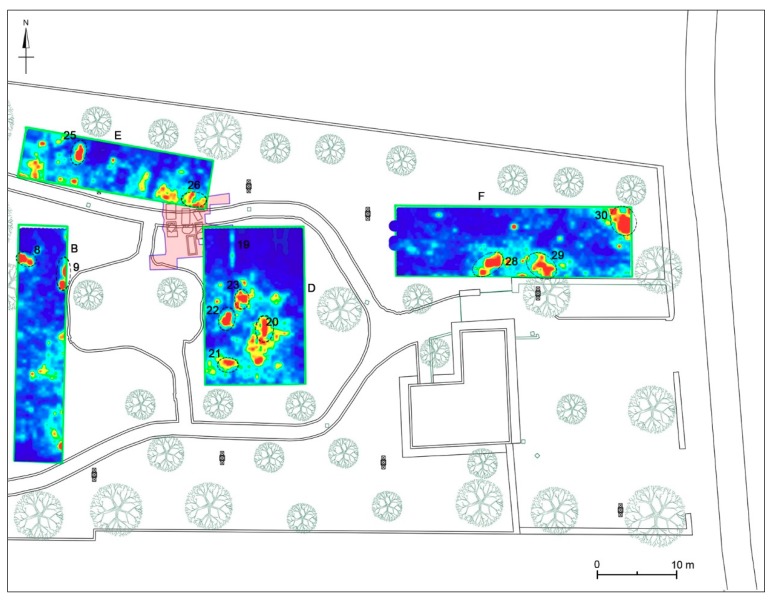
Western sector of the investigated area. B: time-slice at 101–124 cm depth; D: time-slice at 68–87 cm depth; E: time-slice at 111–130 cm depth; F: time-slice at 123–142 cm depth.

Linear anomalies nos. 13 and 14 (Figure 23), visible in the southern portion of the area between 40 and 70 cm deep, are very interesting. They are arranged to form a right angle and can be referred to two walls of uncertain chronology (related to a square structure?) setting on the rocky bank. In the northern portion of the investigated area, between 85 and 140 cm deep, there are two anomalies (nos. 8–9) oriented respectively in an east-west and north-south direction (Figure 24): they are referable to cavities, which according to their shape and size can be identified with other tombs made of stone slabs or sarcophagi.

Area C (m 11 × 14.50; 159.50 m^2^) is immediately west of Area B and it is partly superimposed on the area investigated in 1982 and now buried (see Section 3.2). Between 15 and 60 cm deep there is an anomaly (no. 18) elongated in a north-west/south-east direction and referable to a cavity (Figure 20 and Figure 21). It is located on the same alignment of the tombs made of chests of stone slabs nos. 1–4 and 6, discovered in 1981, and could be identified with another tomb of the same group (and type?). The same interpretation could be proposed for anomaly no. 17, stretched in a north-south direction, visible a little further to the east between 40 and 70 cm deep (Figure 20 and Figure 21). Moreover, two large anomalies (nos. 15–16) are visible in the northern portion of the area (Figure 22): they are documented between 70 and 130 cm deep, up to the bedrock (which in the investigated area is already attested at –110 cm) and correspond to two sectors of the 1982 excavation; in particular, the anomaly no. 15 corresponds in part to the tomb no. 5/82 (see Section 3.2), and partly maybe related to its excavation, and perhaps to a second tomb very close, just outside the excavated area.

#### 4.4.2. Areas D-G

Area D corresponds to a rectangular area of 12.5 × 19.5 m (244 sqm) located east of Area B and partly superimposed (with the north-west corner) to a small portion of the area investigated by archaeological excavations during 1985 and now buried (see Section 3.2). The georadar measurements showed that in the central and southern sector the bedrock is already at 90–100 cm deep, while towards the north it tends to deepen up to 140–150 cm. No anomalies referable to the area excavated in 1985 are noted, with the exception of a linear track oriented in a north-south direction (no. 19), visible from 15 to 130 cm deep and placed at the limits of the archaeological excavation area (Figure 20 and Figure 23). The most significant anomalies (nos. 20–23), instead, concentrate in the central portion of the area and correspond to cavities documented between 40 and 90 cm deep (Figure 20, Figure 23 and Figure 24): for dimensions and orientations (east-west and north-south), coherent with those of the nearby tombs brought to light in 1985, they could be chests of stone slabs or sarcophagi.

Area E is extended m 6 × 24 (144 m2) and is located north of Area B. Its eastern portion is superimposed on the area excavated in 1985, today buried (see Section 3.2). In the eastern part of the area, the bedrock is already attested at a depth of 60 cm, deepening towards east and north-east. Between 30 and 90 cm of depth two anomalies (nos. 24–25) referable to cavities are visible (Figure 20), which due to their size could be identified with chests of stone slabs or sarcophagi, similar to those of the near excavated area. Also anomalies nos. 24 and 25 are oriented in a north-south direction as many of these tombs. Some anomalies at between 90 and 140 cm depth can be reported, including the one (no. 26), perhaps related to the two northernmost tombs brought to light in 1985 (Figure 23 and Figure 24).

**Figure 25:**
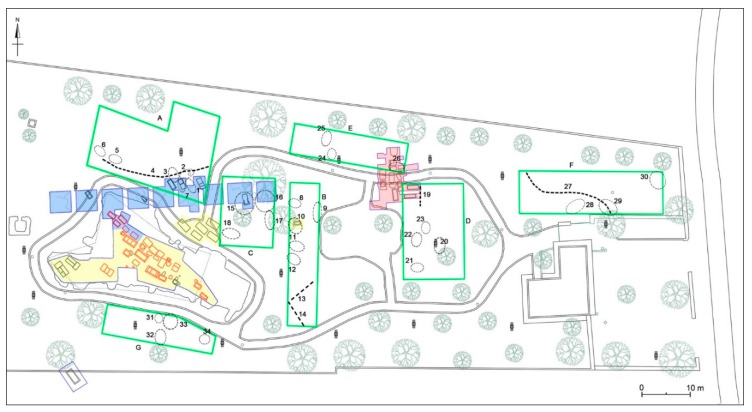
Archaeological map of the Monte D’Elia necropolis. It is possible to highlight the distribution the anomalies related to probably new tombs with the georeferenced with respect to the already excavated tombs.

Area F, extending over an area of 9 × 31.5 m (about 284 m2), is at the north-eastern end of the archaeological park, in an area where no archaeological evidence is reported. This area is crossed by a modern pipeline, as documented by a very evident anomaly (no. 27) visible between 40 and 80 cm deep (Figure 20). Starting from 80 cm of depth, in the southern and eastern part of the area is already attested the bedrock. Here, between cm 90 and 150 deep, three anomalies (nos. 28–30) are visible (Figure 24 and Figure 25), which can be identified with cavities dug into the rock and can be interpreted as other tombs filled with soil and stones.

Area G has an irregular shape of about 23 × 6 m (about 126 m^2^) and is immediately south of the funerary area unearthed in 1981 (see Section 3.2). The bedrock is attested between 80 and 135 cm deep. Between 40 and 90 cm of depth two anomalies (nos. 31–32) are evident (Figure 20), oriented in a north-south direction and referable to cavities, which could be identified with chests of stone slabs or sarcophagi. Between 70 and 115 cm of depth we can see another anomaly (no. 33) with the same characteristics, but, in consideration of the dimensions, it could have been produced by two tombs side by side (Figure 21). Finally, another anomaly (no. 34) possibly reliable to a tomb can be seen between 100 and 145 cm deep at the eastern edge of the area (Figure 22). It is partially embedded in the rocky bank. If the interpretation of these anomalies is correct, it would confirm the extension to the south of the funerary area brought to light in 1981. Also the depths to which the burials would be found are coherent with those of the area investigated by archaeological excavations.

## 5. Conclusions

The topographical and geophysical surveys carried out in the area of the Messapian necropolis of Monte D’Elia allowed for the collection of important new data on the extension of the funerary area and its topographical organization. In particular, topographical surveys allowed for the production of a new map of the overall necropolis, where the old plans of the tombs unearthed during the 1981–1985 archaeological excavations were georeferenced. In this new plan, thanks to the comparison with the grave goods studied in the 1980s–1990s and the examination of the types of burials, it was possible to distinguish the tombs of the Archaic period and those of the Classical/Hellenistic age, which partly overlap the oldest ones.

The geophysical prospecting allowed to highlight numerous anomalies, many of them with archaeological interest, in part related to the tombs brought to light in 1981–1985 and subsequently buried, in part to new tombs (around 25), unknown and yet to be explored. The identification of the new tombs was possible thanks to the georeferencing of the time slices from GPR investigations in the new archaeological map of the necropolis; moreover, the visualization of these anomalies in the virtual environments of the subsoil, produced thanks to the integration of the 3D topographical and geophysical data, was very useful in the interpretation phase (Figure 25).

The georadar measurements allowed us to document the extent of the necropolis beyond the limits that were defined at the time of archaeological excavations, both north and south, and especially to the west. In addition, by integrating the plan of archaeological investigations and the results of geophysical prospecting it is possible to retrieve some data on the articulation of the funerary area, only partly conceivable thanks to the archaeological excavations of 1981–1985. In particular, in the western sector the tombs seem predominantly oriented in a north-west/south-east direction in relation to a road crossed the necropolis with the same direction. In the eastern sector, on the other hand, the graves seem to have a predominantly north-south orientation, with some burials arranged orthogonally in an east-west direction. In this case, it could be assumed that this sector of the necropolis was influenced by another road (maybe the ancient Via Sallentina) that ran nearby with a north-south orientation. Lastly, there are not large (for example 2 × 3 m or more) anomalies attributable to chamber tombs; at the current state of research, this result seems to confirm the previous hypothesis on the absence of this type of tomb in the necropolises of Aletion/Aletium (D’Elia 2001, p. 20) according to the archaeological evidence, contrary to the situation documented in other Messapian settlements, such as Rudiae, Vaste, Egnatia and Lecce.

In conclusion, it can be highlighted how the results of the topographical and geophysical surveys at necropolis of Monte D’Elia not only allowed us to collect new data for the knowledge of this important funerary area and its organization, but also for planning future stratigraphic excavations. Indeed, on the one hand, archaeological excavations could focus the anomalies probably linked to new tombs in order to constitute a verification of the geophysical prospecting results. On the other hand, these excavations can increase the knowledge of the necropolis and reconstruct its use over the centuries, allowing for the recovery of further grave goods for a better chronological definition of the use of the funerary area and, more generally, increasing the knowledge of the socio-economical aspects of Aletion/Aletium during the Messapian era, such as material culture, artisan productions, imports from the Greek world, etc.
